# A Highly Specific Antibody-Based Assay for *Nipah* Virus AlphaLISA Detection

**DOI:** 10.3390/v17060748

**Published:** 2025-05-23

**Authors:** Xuyang Sun, Qingyu Lv, Wenhua Huang, Xinran Zhang, Huiqi Duan, Yuhao Ren, Xiaojing Zhang, Yongqiang Jiang, Ruili Zhao, Shaolong Chen

**Affiliations:** 1 Tianjin Key Laboratory of Agricultural Animal Breeding and Healthy Husbandry, College of Animal Science and Veterinary Medicine, Tianjin Agricultural University, Tianjin 300392, China; 17610852833@163.com; 2 State Key Laboratory of Pathogen and Biosecurity, Academy of Military Medical Sciences, Beijing 100071, China; lvqingyu2004@126.com (Q.L.); huangwh1993@163.com (W.H.); 13820530560@163.com (X.Z.); d1449009136@163.com (H.D.); www2354@126.com (Y.R.); 18047114491@163.com (X.Z.); jiangyq@bmi.ac.cn (Y.J.)

**Keywords:** nipah virus, monoclonal antibodies, single B cell antibody production technology, AlphaLISA, virus detection

## Abstract

Nipah virus (NiV) is an emerging zoonotic pathogen whose surface glycoprotein (G)-mediated host cell invasion mechanism leads to fatal encephalitis in infected patients (case fatality rate 40–75%). Given the limitations of existing diagnostic technologies, such as low sensitivity and prolonged processing times, we prepared an anti-NiV-G monoclonal antibody to establish a novel Amplified Luminescent Proximity Homogeneous Assay (AlphaLISA) detection system. Firstly, five high-affinity anti-NiV-G monoclonal antibodies were screened from the spleens of immunized mice by flow cytometry-single-cell cloning technology. The reaction system was further optimized, and the optimal dilution ratio of antibody-conjugated receptor microspheres, biotinylated antibodies, and donor microspheres was screened, and the AlphaLISA detection platform was successfully constructed. The detection sensitivity of NiV-G protein was 0.024 ng/mL (41.7 times higher than that of conventional ELISA), the coefficient of variation was <9.5%, and the repetition was good. It showed good specificity in the detection of 5 zoonotic viruses, including Japanese encephalitis virus and Zika virus. At the same time, this method is less disturbed by human serum, and the detection time is less than 30 min, showing a good clinical application prospect.

## 1. Introduction

Nipah virus (NiV) is a zoonotic pathogen newly discovered in Malaysia in 1998 and belongs to the family *Paramyxoviridae* and *Henipavirus genus*, and its infection can cause severe neurological and respiratory disease and fatal encephalitis [1,2]. Since 1998, successive NiV outbreaks have been reported in subtropical regions, including Singapore, Bangladesh, the Philippines, and India, which pose a major threat to public health [3]. Most recently, a new outbreak of NiV was reported in Kerala, India, in August 2023, with 30 cases of infection reported and a fatality rate of 33.6 percent [4]. Nipah virus is recognized by the World Health Organization as a biosafety level 4 (BSL-4) pathogen and is placed on the priority pathogen list [5,6].

The virulence of NiV begins with the fusion of the virus with the host cell membrane. This fusion primarily relies on the virus’s G protein and F protein, which are important glycoproteins that facilitate the binding of the virus to the ephrinB2 and ephrinB3 receptors on the surface of host cells [7,8]. This binding leads to the fusion of the virus and the cell membrane, promoting the invasion of the virus into the cells. The NiV-G protein consists of an N-terminal transmembrane domain, a neck domain, and a C-terminal receptor-binding head domain [9]. Furthermore, even when only the head domain is present, it still exhibits high specificity and immunogenicity, which is why the NiV-G protein serves as the primary immunodominant target for neutralizing antibodies against NiV [10]. Therefore, the NiV-G protein is frequently utilized in the development of vaccines and diagnostic antibodies against NiV [11].

Since Kohler and Milstein pioneered the generation of monoclonal antibodies (mAbs) via hybridoma technology in 1975, these antibodies have emerged as indispensable research tools and potent therapeutic agents. [12]. However, classical hybridoma techniques are constrained by a PEG-mediated random fusion process, which typically results in an effective fusion rate of only 1 × 10^−6^ to 1 × 10^−5^ (i.e., 1 to 10 stable antibody-secreting hybridoma cells per million lymphocytes), with a B-cell-specific fusion success rate of less than 5 × 10^−7^ [13]. Consequently, these methods are unable to rapidly respond to the emergence of new viral threats [14]. In recent years, single-B cell antibody screening technology has emerged as a promising alternative due to its ability to efficiently screen and clone antibodies targeting specific antigens directly from recovered or immunized animals [15]. This platform preserves the natural pairing of heavy and light chains by passing the inefficient hybridoma fusion step, thereby enabling more efficient exploration of immune B cell populations [16]. Although significant progress has been made in applying single-B cell antibody screening technology to various viruses, there remains a paucity of studies focused on the development of monoclonal antibodies and their application in Nipah virus detection methods. Moreover, existing Nipah virus detection methods exhibit limitations in terms of sensitivity and operational simplicity. Therefore, further exploration into the development of technologies based on high-quality monoclonal antibodies for Nipah virus detection is urgently required.

Laboratory diagnostic methods for Nipah virus are mainly divided into serological diagnostic methods and molecular diagnostic methods. Traditional serologic diagnostic methods include enzyme-linked immunosorbent assay [17]. ELISAs susceptibility to aerosol contamination during manual handling, limited sensitivity (ng/mL range), and cross-reactivity with phylogenetically related henipaviruses render it suboptimal for robust Nipah virus detection, particularly in resource-limited settings facing high genetic diversity strains. Molecular diagnostic methods include a range of detection methods for viral nucleic acids, including real-time fluorescence reverse transcription-polymerase chain reaction (qRT-PCR), multiplex PCR, reverse transcription PCR, and dual-panel RT-PCR, as well as a variety of nucleic acid sequencing methods in addition to traditional PCR methods [18]. Among them, RT-PCR is the preferred method for high-throughput testing, but the time required from sampling to generating test results is usually several hours. In addition, qRT-PCR requires specialized equipment, such as thermal cyclers, and skilled technicians [19]; however, Nipah virus is more commonly found in backward areas, which may affect the sensitivity and specificity of qRT-PCR detection, so the occurrence of Nipah virus outbreaks cannot be detected quickly. Therefore, there is a need to develop a viral detection method that is as sensitive and rapid as qRT-PCR in order to promote NiV detection in endemic areas at risk of outbreaks.

AlphaLISA is a chemiluminescence-based homogeneous immunodetection technique wherein streptavidin-coated donor microspheres are excited by a 680 nm laser. This excitation induces the release of singlet oxygen, and if the antibody is positioned within 200 nm, the amplified fluorescent signal in the receptor microsphere is activated, generating light emission at 615 nm [20,21]. In comparison to ELISA, AlphaLISA eliminates the requirement for multiple intricate manual wash steps to separate bound and unbound components, thereby enhancing assay simplicity and reducing overall assay time [22]. Based on these principles, AlphaLISA exhibits the advantages of being wash-free, highly sensitive, and possessing a broad signal dynamic range [23], making it widely applicable in disease marker detection and drug screening research, particularly for the early diagnosis of infectious diseases.

**Figure 1:**
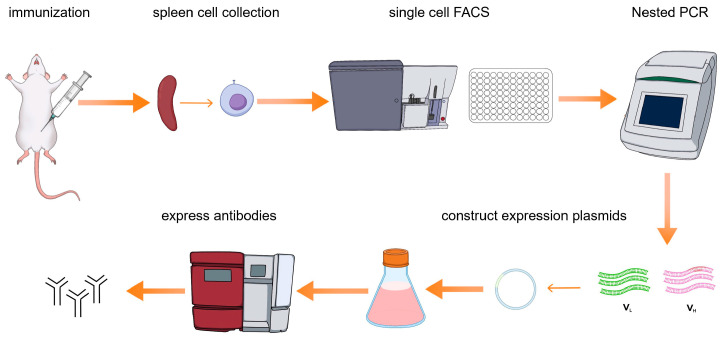
Workflow summary of mAb discovery pipeline in mice. Mice were immunized via intraperitoneal injection following a standardized protocol. Spleens were harvested post-immunization and processed into single-cell suspensions. After staining the cell suspension, NiV-positive specific memory B cells were sorted into 96-well plates by flow cytometry and lysed, and then the RNA encoding the monoclonal antibody was reverse transcribed, PCR amplified, and cloned into the expression plasmid, and the antibody was expressed using the Expi293 transient transfection system, and the antibody was purified using a Protein A column to obtain an NiV monoclonal antibody.

Here we present a single B cell screening platform utilizing multicolor fluorescence-activated cell sorting (FACS) to isolate and generate NiV-G-specific monoclonal antibodies (mAbs) from immunized mice (Figure 1). The antibodies generated through this approach serve as highly efficient biological reagents, enabling direct application to downstream diagnostic assays without requiring chemical modifications or structural optimization. This methodology not only streamlines antibody production but also establishes a foundation for clinical development. By leveraging high-affinity NiV-G monoclonal antibodies, we further developed an AlphaLISA assay for the detection of Nipah virus (NiV). Through the optimization of the AlphaLISA method, its advantages in detection sensitivity, specificity, and anti-interference were evaluated. The establishment of the AlphaLISA detection method will provide strong technical support for the early diagnosis and epidemic prevention and control of Nipah virus infection and provide ideas and references for the detection of other emerging infectious diseases.

## 2. Materials and Methods

### 2.1. Collection of Spleen Cells from Immunized Mice

Six-week-old male BABL/c mice were acquired from Beijing Speufort Biotechnology Co., Ltd (Beijing, China). for this study, with all immunizations administered via intraperitoneal injection using a standardized protocol. The initial immunization (Day 0) consisted of 150 μL NiV-G protein (50 μg/dose, Sino Biological, Beijing, China) emulsified with Freund’s complete adjuvant (Sigma-Aldrich, St. Louis, MO, USA), followed by three subsequent immunizations at 21-day intervals using identical antigen quantities combined with Freund’s incomplete adjuvant (Sigma-Aldrich, St. Louis, MO, USA). Tail vein blood samples (150 μL) were collected seven days after the third immunization and again following the fourth dose, with all spleen collections performed two days after the final immunization. Animals were humanely euthanized at this timepoint, and spleens were processed within 24 h of collection to maintain cellular viability: splenocytes were isolated through mechanical disruption using a 40 μm cell sieve (Biologix, Jinan, China) pre-coated with HBSS containing 20% fetal bovine serum, followed by centrifugation (1500× *g*, 5 min) and resuspension in flow cytometry buffer (calcium/magnesium-free HBSS supplemented with 1% FBS, 1 mM EDTA, and 25 mM HEPES).

### 2.2. Isolation of NiV-G Protein-Specific Memory B Cells

A single-cell suspension was prepared from murine splenocytes of NiV-G protein-immunized animals and adjusted to 1 × 10^7^ cells/mL (500 μL total volume). Cellular staining was performed using a multicolor antibody panel, with experimental groups receiving dual labeling through APC and PE-conjugated NiV-G recombinant protein. Isotype controls underwent single-color compensation staining. All incubations were executed in the dark at 4 °C with periodic vortex mixing to ensure homogeneous antibody binding. Flow cytometric sorting was conducted on a BD FACS Aria III system (BD Bioscience, CA, USA) utilizing a 100 μm nozzle and 20 psi sheath pressure, with sorting events restricted to ≤2000/s to preserve viability. Live cell discrimination was achieved through 7-AAD incorporation (final concentration 2%) added five minutes prior to acquisition, ensuring >95% viability in the 7-AAD-negative population. Gating analysis was implemented via BD FACSDiva 8.0.1 software following three-step stratification: lymphocyte identification (FSC-A/SSC-A parameters, 62.3 ± 4.1%), singlet selection (FSC-W/FSC-H discrimination, <1.2% doublets), and B cell subset isolation (CD19 + IgG + IgM-IgD-population constituting 8.7 ± 1.3% of lymphocytes). Antigen-specific B cells (0.15 ± 0.03% of total B cells) were sorted into 96-well PCR plates containing 5 μL lysis buffer per well, followed by immediate cryopreservation at −80 °C in liquid nitrogen.

### 2.3. Amplification of the Coding Sequence of the Antibody

Lysed B cells underwent reverse transcription PCR using Aomei Biotechnology (Beijing, China) reagents to generate cDNA following manufacturer specifications. Antibody variable region genes were amplified through a two-round nested PCR strategy utilizing primers designed based on von Boehmer et al. [24] (specific sequences in Table S1). Reaction systems (40 μL) contained 2 μL cDNA, 0.4 μM forward/reverse primers (Table S1), 20 μL 2× M5 Hiper plus Taq HiFi mix (Mei5 Biotechnology, Beijing, China), and ddH_2_O. Thermal cycling parameters included initial denaturation (95 °C, 3 min), 35 cycles (94 °C/25 s → 57 °C/25 s → 72 °C/25 s), and final extension (72 °C, 5 min). Amplified products (~300 bp light chain, ~500 bp heavy chain) were verified by 1% agarose gel electrophoresis (120 V, 30 min). Second-round nested PCR enhanced specificity through V gene 5’ high-variable region forward primers and constant region reverse primers with optimized conditions (0.2 μM primers, 20 s annealing). Validated amplicons were purified and commercially sequenced (Sangon Biotech, Shanghai, China). Variable region sequences were directionally cloned into pcDNA3.1(+) containing murine IgG1 heavy chain and κ light chain constant regions via human codon optimization, with sequence validation followed by cryopreservation.

### 2.4. Expression and Purification of Monoclonal Antibodies

Co-transfection of light chain and heavy chain expression plasmids into Expi293 cells (2 × 10^6^ cells/mL, logarithmic phase) was performed using Beijing Mei5 Biotransfection Reagent protocols, followed by 7-day incubation at 37 °C with 8% CO_2_ and 120 rpm orbital shaking. Culture supernatants were harvested by centrifugation (10,000× *g*, 4 °C, 10 min), sterilized through 0.22 μm PES filtration (Millipore, Billerica, MA, USA), and subjected to Protein A affinity chromatography purification using an ÄKTA pure system (Cytiva, Co., LLC., Marlborough, MA, USA). HiTrap Protein A HP columns (5 mL) were equilibrated with 20 mM sodium phosphate buffer (pH 7.4, 150 mM NaCl) across five column volumes, loaded at 3 mL/min with ≤20 mg IgG/mL resin capacity, and eluted via a pH 3.5 sodium acetate gradient (0–100% elution buffer over 10 column volumes). Fractions were immediately neutralized with 1 M Tris-HCl (pH 8.5) to 50 mM final concentration, then dialyzed against PBS (pH 7.4) for 12 h at 4 °C using Slide-A-Lyzer™ 10 K MWCO devices (Thermo Fisher, Waltham, MA, USA) with four-hour interval buffer exchanges. Antibody titers were quantified by indirect ELISA (NiV-G coating at 2 μg/mL, HRP-conjugated secondary antibody diluted 1:8000) and the OD450 was measured using a SpectraMax™ I3 microplate reader (Molecular Devices, Co., LLC, Sunnyvale, CA, USA). Target protein purity and molecular weight were confirmed through reduced SDS-PAGE analysis (12% separating/5% stacking gels: 80 V initial → 120 V final until bromophenol blue migration) combined with Coomassie brilliant blue R-250 staining and destaining protocols.

### 2.5. Preparation of Components for AlphaLISA Assay

The five Nipah virus monoclonal antibodies (NiV-6, NiV-8, NiV-12, NiV-22, and NiV-63) identified through indirect ELISA screening underwent biotinylation individually. Antibody concentrations were standardized to 1 mg/mL using pH 7.0 PBS, followed by the addition of 2 μL Sulfo-NHS-LC-Biotin (10 mM stock solution, Thermo) to 100 μL antibody solutions. Reactions were conducted at 25 °C in constant-temperature rotating mixers (600 rpm, 1 h), with free biotin removed via Zeba™ Spin desalting columns (7K MWCO, Thermo) (centrifuged at 1500× *g* for 2 min). Biotinylated antibodies were stored at 4 °C until subsequent coupling procedures. Receptor microspheres (aldehyde-functionalized, PerkinElmer, Inc, Waltham, MA, USA) were then conjugated with 75 μg antibody per 100 μL reaction volume containing 5 μL 0.4 M sodium cyanoborohydride and 0.625 μL 10% Tween 20, adjusted to 100 μL final volume with 100 mM HEPES buffer (pH 7.4). Reactions proceeded under dark conditions at 37 °C in rotating mixers for 24 h, followed by the addition of 5 μL carboxymethoxyamine (65 mg/mL) for 1 h incubation. Unbound components were removed through three centrifugation-wash cycles (16,000× *g*, 15 min, 0.1 M Tris-HCl buffer pH 8.0). Conjugated microspheres were resuspended in PBS (pH 7.0) containing 0.05% Proclin-300, sonicated (20 kHz, 20 W, 30 s), and stored at 4 °C.

### 2.6. The Procedure of the AlphaLISA Detection Method

The AlphaLISA detection principle employs a sandwich immunoassay format utilizing a two-step measurement protocol. A 20 μL reaction mixture containing receptor microsphere-labeled NiV antibodies and biotinylated NiV antibodies was prepared in AlphaLISA buffer (50 mM HEPES pH 7.4, 0.2% casein, 2 mg/mL dextran-500, 0.1% Triton X-100, 0.1% Proclin-300), with volume adjustments made using PerkinElmer Inc (Waltham, MA, USA). reagents. Detection samples (NiV-G protein or Nipah virus pseudovirus) were diluted and added to wells (10 μL/well), followed by incubation at 37 °C for 15 min with orbital shaking. Streptavidin-conjugated donor microspheres (PerkinElmer Inc, Waltham, MA, USA) were then added (20 μL/well) and incubated under dark conditions at 37 °C for 10 min. Fluorescence intensity at 615 nm was measured using a SpectraMax™ I3 microplate reader (Molecular Devices, Co., LLC, Sunnyvale, CA, USA). Analytical parameters were established through calculation of the critical value (mean fluorescence of negative controls + 3 × standard deviation) and determination of signal-to-noise ratios (test sample fluorescence/critical value).

### 2.7. Optimization of the AlphaLISA Detection Method System

Following the experimental protocol established by Huijun Zong et al. [22], biotinylated antibodies underwent 4000-fold dilution, receptor-coupled microspheres were diluted 200-fold, and donor microspheres were initially diluted 250-fold. AlphaLISA detection was performed using 100 ng/mL NiV-G protein with paired antibody combinations. Antibody pairs demonstrating the highest signal-to-noise ratios (S/N) were identified as optimal for subsequent optimization. Antigen concentration was standardized to 10 ng/mL Nipah virus pseudovirus under fixed reaction conditions (37 °C, 10 min). A three-phase optimization strategy was implemented: Donor microsphere optimization: Biotinylated (4000-fold) and receptor-coupled (200-fold) antibodies were maintained at fixed concentrations while donor microspheres underwent continuous 2-fold serial dilution (200- to 12.5-fold). S/N ratios were systematically compared across dilution gradients. Biotinylated antibody optimization: Following donor microsphere optimization, receptor-coupled antibody dilution (200-fold) was fixed. Biotinylated antibodies were serially diluted 2-fold (16,000- to 1000-fold) to identify the dilution yielding maximum S/N. Receptor microsphere validation: With confirmed donor and biotinylated antibody parameters, receptor-coupled microspheres underwent 2-fold serial dilution (3200- to 100-fold). S/N ratios were compared to validate the optimal receptor dilution.

The highest S/N value from each phase was selected as the definitive dilution parameter for subsequent experiments. All dilution series were performed in triplicate to ensure reproducibility.

### 2.8. Test Sensitivity

The NiV-G protein was serially diluted in a 2-fold gradient (100 ng/mL to 0.012 ng/mL) and analyzed via AlphaLISA. Triplicate measurements were performed for each concentration to establish the standard curve model for NiV-G detection. Simultaneously, *Nipah* pseudovirus (Sino Biological, Inc., Beijing, China) was serially diluted from 9450 TU/mL (2-fold increments) to 18.5 TU/mL and similarly analyzed by AlphaLISA with three technical replicates per concentration to generate the pseudovirus standard curve. The critical value was defined as the mean absorbance of negative controls plus three standard deviations, with signal-to-noise ratios calculated as the ratio of each concentration’s absorbance to this critical value. The detection limit was established at 1, with the minimum detectable concentration defined as the lowest dilution exhibiting a signal-to-noise ratio exceeding 1.

### 2.9. Test Specificity

To assess the specificity of the AlphaLISA detection, five viruses similar to the Nipah virus in structure, pathogenicity, and susceptible receptors (Zika virus, influenza A virus, influenza B virus, Japanese encephalitis virus, and *coxsackievirus*) were selected for AlphaLISA detection to evaluate their specificity. The NiV-G antigen and five additional viral antigens were serially diluted to three gradient concentrations (100 ng/mL, 10 ng/mL, and 1 ng/mL), with three technical replicates established for each virus and negative control group. The standard deviation was calculated from triplicate absorbance measurements, and the cutoff value was defined as the mean absorbance of negative controls plus three standard deviations. Signal-to-noise ratios (S/N) were determined by dividing each concentration’s absorbance by the cutoff value, with detection limits set at an S/N threshold of 1. Positive results were defined as S/N values exceeding 1.

### 2.10. Test Repetitive

Three different concentrations (10 ng/mL, 1 ng/mL, and 0.1 ng/mL) of NiV-G protein were used to verify the repeatability of AlphaLISA. A total of 3 independent experiments were performed in 3 replicate wells for each concentration to verify the inter-assay coefficient of variation (CV) of AlphaLISA; in one experiment, 12 replicate wells were detected at each concentration to verify the detection of intra-assay CV.

### 2.11. Test Anti-Interference

Porcine serum (10%) and human serum were utilized as background matrices for AlphaLISA anti-interference testing. Simulated samples containing NiV-G protein or *Nipah* pseudovirus were prepared by spiking serially diluted analytes into buffer-serum mixtures. Antigen concentrations were serially diluted 2-fold (100 ng/mL → 0.012 ng/mL for NiV-G; 94,500 TU/mL→93 TU/mL for pseudovirus) with three technical replicates per concentration. Negative controls consisted of buffer-serum mixtures without analyte addition. The cutoff value was defined as the mean absorbance of negative controls plus three standard deviations, with signal-to-noise ratios calculated as the ratio of each concentration’s absorbance to this cutoff. The detection limit was established at 1, with the minimum detectable concentration defined as the lowest dilution exhibiting a signal-to-noise ratio exceeding 1.

### 2.12. Statistical Analysis

This study’s experiments were repeated three times. Statistical analysis of all data were conducted using GraphPad Prism 8.0, SPSS 26.0, and SigmaPlot 14.0 software. Group comparisons were analyzed using one-way ANOVA followed by Dunnett’s post-hoc test using GraphPad Prism 8.0. The optimal cutoff value was determined when the Youden index reached its maximum value. A *p*-value of <0.05 was considered statistically significant.

## 3. Results

### 3.1. Isolating Highly Specific NiV-G + Memory B Cells

**Figure 2:**
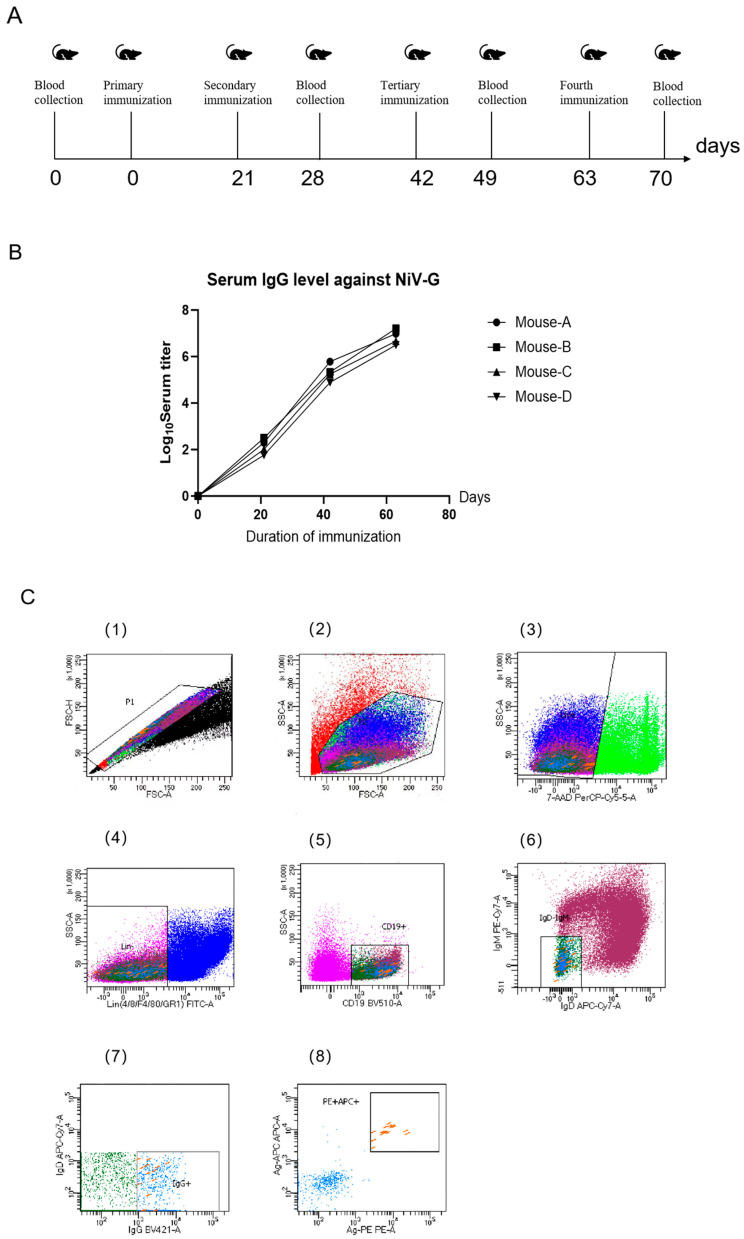
Comprehensive Evaluation of the Immune Response of BALB/c Mice to NiV-G Protein: Immunization, Determination of Serum IgG Titers, and Sorting of Antigen-Positive B Cells. (**A**) Immunization and blood collection process of BALB/c mice. (**B**) The titer of IgG anti-NiV-G protein in mouse serum was determined by indirect ELISA, and the data were the average of three serum titers. (**C**) Sorting of NiV antigen-positive B cells. Splenocytes were collected from immunized mice, B cell enrichment, and stained for FACS analysis (1,2): TruStain FcX reagent blocks lymphocytes after non-specific Fc receptor deadhesion (3): 7AAD was selected to remove dead cells (4): FITC negative was selected sequentially to exclude T cells to select B cells (5): BV510 was positive, CD19+ was the marker molecule of memory B cells, and IgG was expressed on the surface of memory B cells through CD19 + Memory B cells expressing IgG were co-screened with IgG fluorescent monoclonal antibody (6): PE-Cy7 negative and APC-Cy7 negative (7,8) were selected in order. Cell populations with positive antigens for PE and APC were screened. Finally, this cell population accounted for less than 0.01%, and a total of 288 phenotypic titer memory B cells were screened from three 96-well plates.

Six-week-old BALB/c female mice were immunized with NiV-G protein to obtain memory B cells specific for Nipah virus. The immunization schedule is shown in Figure 2A. The level of humoral immune response in mice was evaluated by the indirect ELISA method. As shown in Figure 2B, the immune titers of all four mice after the third immunization could reach 1:10^5^ (log10 = 5.0 ± 0.8). After the fourth immunization, the immune titers of the four mice were significantly increased, among which Mouse-A and Mouse-B mice showed a higher level of immune response to NiV-G protein, and Mouse-B mice had the highest immune titer of 1:10^7^ (log10 = 7.2). Therefore, the spleens of these two mice were taken to isolate splenocytes for subsequent single B cell sorting. As shown in Figure 2C, a total of 288 phenotypic titer memory B cells were screened by screening memory B cells co-expressed with CD19+ and IgG+ fluorescent monoclonal antibodies, and then PE- and APC-labeled antigen double-positive cell populations were selected sequentially, and the cell population accounted for less than 0.01%. The results demonstrated that this method efficiently identified and isolated a substantial population of antigen-specific B cells from the spleens of immunized mice.

### 3.2. Obtaining the Variable Region Genes of Antibodies

**Figure 3:**
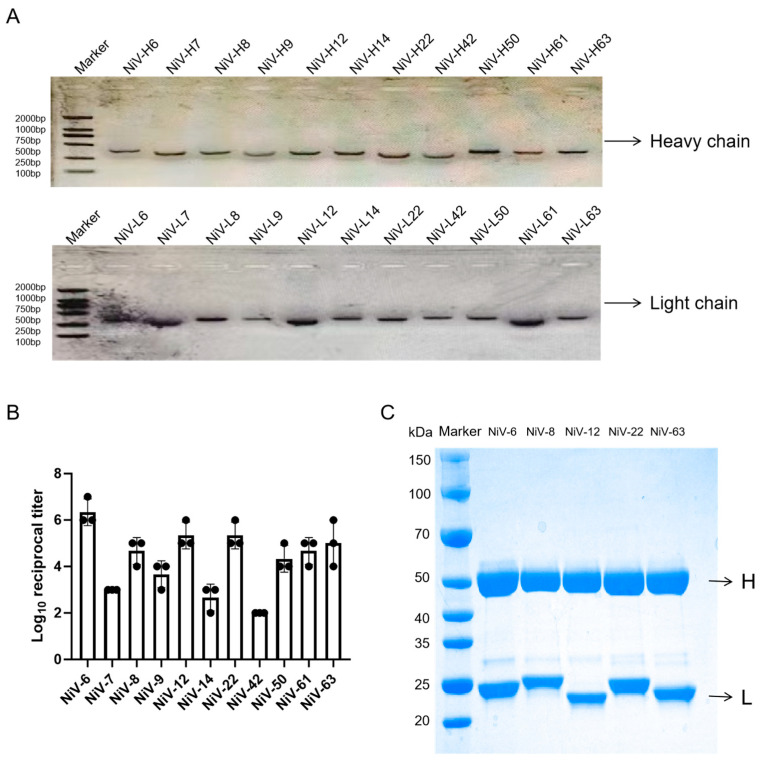
Expression and Identification of Monoclonal Antibodies. (**A**) Single-cell PCR amplification antibody variable region gene. Marker: DNA molecular standard quality Heavy chain: antibody heavy chain; Light chain: antibody light chain. (**B**) Indirect ELISA method to determine antibody-antigen binding affinity; data are the average value of three mouse serum titers. (**C**) SDS-PAGE electrophoresis of purified antibody. Marker: DNA molecular standard quality H: antibody heavy chain L: antibody light chain.

The nested PCR strategy was employed to stepwise amplify the antibody variable region genes. The amplification products were analyzed by 1% agarose gel electrophoresis (120 V, 30 min), revealing that the heavy chain variable region (VH) bands were concentrated at 500 ± 25 bp, while the light chain variable region (VL) was at 300 ± 15 bp (Figure 3A), which is consistent with the theoretical lengths predicted for mouse-derived IgG variable regions in the IMGT database (VH: 480–520 bp, VL: 280–320 bp). Systematic amplification of 288 single-cell samples yielded 116 positive clones for the VH gene (40.2%) and 158 positive clones for the VL gene (54.8%), successfully obtaining 69 pairs of complete VH/VL paired sequences (23.9%). All paired sequences were verified by Sanger sequencing to contain complete FR1-FR4 framework regions and complementarity-determining regions (CDR1–3), with 87% of the sequences (60/69) confirmed to meet the characteristics of functional rearrangement (no frameshift mutations/stop codons). Ultimately, 23 pairs of VH/VL genes were randomly selected for recombinant expression.

### 3.3. Acquisition of Monoclonal Antibodies Against Nipah Virus

This study utilizes purified Nipah virus G protein as a coating antigen and conducts preliminary screening of cell supernatants harvested in small quantities after transfecting 23 constructed cell lines using indirect enzyme-linked immunosorbent assay (ELISA) technology. The experimental results indicate that 16 monoclonal antibodies exhibit specific binding activity to NiV-G protein (Figure S1). Based on the potency assessment criteria (with the potency threshold set at 1:100), 11 high-affinity antibodies (NiV-6, NiV-7, NiV-8, NiV-9, NiV-12, NiV-14, NiV-22, NiV-42, NiV-50, NiV-61, and NiV-63) were selected for large-scale antibody purification. Subsequently, systematic antigen-binding activity analysis of the purified monoclonal antibodies was performed through quantitative ELISA. Experimental data indicated that five antibodies, NiV-6, NiV-8, NiV-12, NiV-22, and NiV-63, exhibited significant antigen-binding activity, with NiV-6 showing a half-maximal binding efficacy (EC50) of up to 1:10^7^ (as shown in Figure 3B), indicating its exceptionally strong antigen affinity. Furthermore, high-affinity antibodies were selected for large-scale purification using Protein A affinity chromatography and were validated by reducing SDS-PAGE electrophoresis (Figure 3C), successfully yielding five high-purity monoclonal antibodies, with heavy and light chain molecular weights of approximately 55 kDa and 25 kDa, respectively, consistent with the typical structural characteristics of IgG antibodies, demonstrating that the purification process effectively maintains the structural integrity and functional activity of the antibodies.

### 3.4. Optimization of the AlphaLISA Detection System

**Figure 4:**
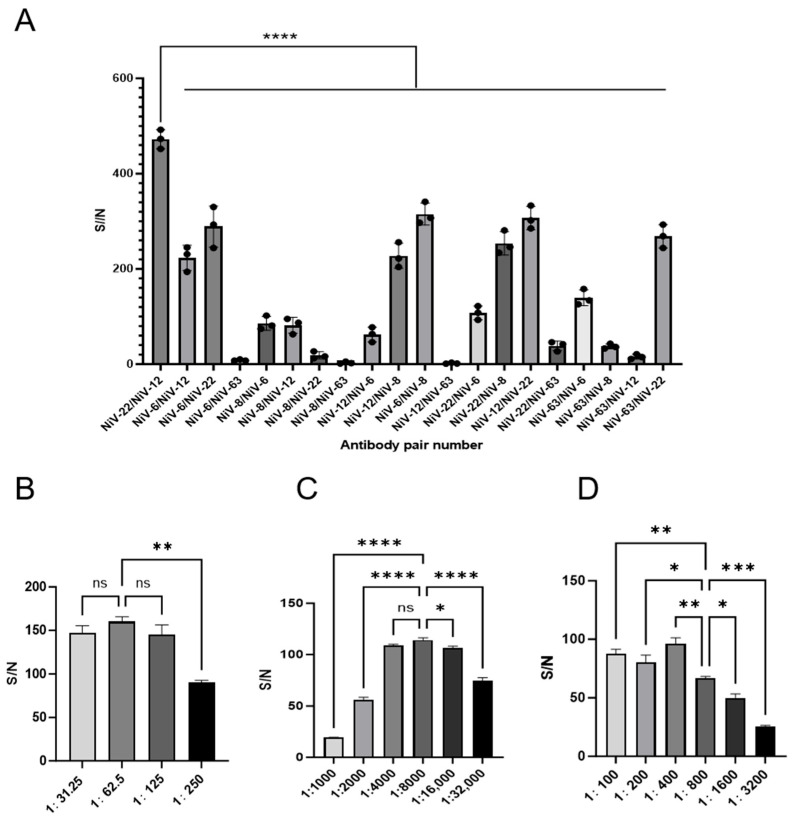
Optimization of AlphaLISA system. ns: no significance, *: *p* < 0.05, **: *p* < 0.01, ***: *p* < 0.001, ****: *p* < 0.0001. (**A**) Screening of the best antibody pairs for the AlphaLISA detection system. (**B**) Optimization of the dilution ratio of donor microspheres. (**C**) Optimization of dilution ratio of biotinylated NiV antibody. (**D**) Optimization of dilution ratio of NiV antibody-conjugated receptor microspheres.

To optimize the detection sensitivity of the Nipah virus based on the AlphaLISA system, this study determined the optimal antibody pairing combinations through orthogonal screening methods. The results showed (Figure 4A) that using the 22nd antibody coupled with the acceptor microsphere and the 12th biotinylated antibody yielded the highest signal-to-noise ratio (S/N). Further optimization of the concentrations of key components was conducted through systematic gradient dilution (Figure 4B–D), revealing that when the biotinylated antibody was diluted 8000-fold (1:8000), the acceptor microspheres were diluted 400-fold (1:400), and the streptavidin-conjugated donor microspheres were diluted 62.5-fold (1:62.5), the detection system achieved optimal performance with low background interference, resulting in the highest signal-to-noise ratio (S/N) and significantly enhanced detection sensitivity.

### 3.5. Sensitivity

**Figure 5:**
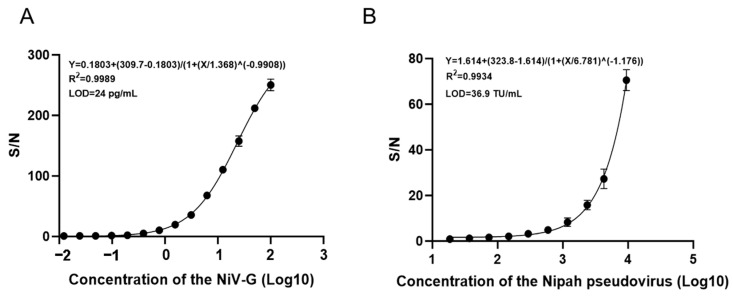
AlphaLISA Sensitivity. (**A**) Standard curve for AlphaLISA detection of Nipah antigen (2-fold serial dilution) (0.012–100 ng/mL). (**B**) Standard curve for AlphaLISA detection of *Nipah* pseudovirus (2-fold serial dilution) (18.5–9450 TU/mL).

Using the AlphaLISA signal-to-noise ratio (S/N) as a function of NiV-G, a standard curve was calculated. The results indicate that the S/N ratio increases with the increase in antigen concentration, with the four-parameter fitting equation being Y = 0.1803 + (309.7 − 0.1803)/(1 + (X/1.368)^(−0.9908)), and the correlation coefficient R^2^ = 0.9989, with a limit of detection (LOD) of 24 pg/mL. (Figure 5A) Similarly, a standard curve was calculated using the Nipah pseudovirus, and the results showed that the S/N ratio increases with the increase in antigen concentration, with the four-parameter fitting equation being Y = 1.614 + (323.8 − 1.614)/(1 + (X/6.781)^(−1.176)), and the correlation coefficient R^2^ = 0.9934, with the LOD of the pseudovirus being 36.9 TU/mL (Figure 5B).

### 3.6. Specificity and Reproducibility

**Figure 6:**
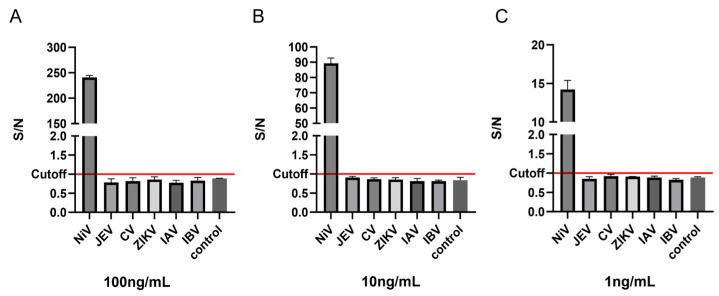
Specificity evaluation of AlphaLISA. (**A**) The S/N values of different viral antigens at 100 ng/mL; (**B**) The S/N values of different viral antigens at 10 ng/mL; (**C**) The S/N values of different viral antigens at 1 ng/mL. NiV: Nipah virus, IAV: Influenza A virus, IBV: Influenza B virus, JEV: Japanese encephalitis virus, CV: Coxsackievirus, ZIKV: Zika virus.

In this study, five zoonotic viruses (ZIKV, IAV, IBV, JEV, CV) were tested. As shown in Figure 6, under the testing conditions of three concentration gradients (1/10/100 ng/mL), the signal-to-noise ratio (S/N) values of the other five viral antigens, except for the Nipah virus (NiV) positive control, remained consistently below the determination threshold of 1, which were determined to be negative results, indicating that this detection system has high specificity. The results of repeatability experiments showed that the intra-assay coefficient of variation (CV = 6.42–8.03) and the inter-assay coefficient of variation (CV = 6.14–9.51) of the three concentration gradients showed that AlphaLISA had good stability (Table 1).

### 3.7. Resistance to Interference

**Figure 7:**
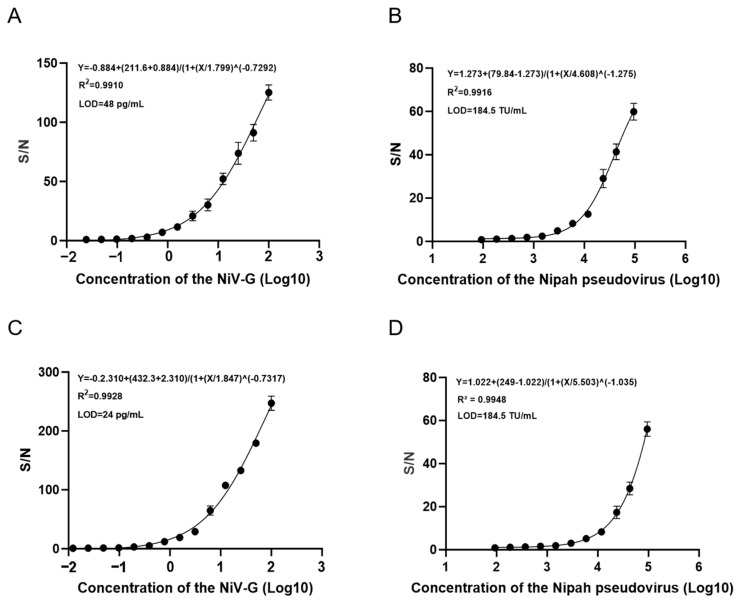
Evaluation of the anti-interference performance of serum using AlphaLISA technology. (**A**) Standard curve (0.024–100 ng/mL) for the detection of porcine serum mixed Nipah antigen (2-fold serial dilution) by AlphaLISA. (**B**) Standard curve (93–94,500 TU/mL) for the detection of porcine serum mixed Nipa pseudovirus (2-fold serial dilution) by AlphaLISA. (**C**) Standard curve (0.012–100 ng/mL) for the detection of human serum mixed Nipah antigen (2-fold serial dilution) by AlphaLISA. (**D**) Standard curve (93–94,500 TU/mL) for the detection of human serum Nipah pseudovirus (2-fold serial dilution) by AlphaLISA.

To assess the interference resistance of the detection system, NiV-G protein and NiV pseudovirus were respectively mixed with 10% human serum and pig serum to simulate real samples, establishing a quantitative standard curve based on the AlphaLISA signal-to-noise ratio (S/N). The results showed that the limit of detection (LOD) of NiV-G protein in pig serum was 48 pg/mL, with the four-parameter logistic (4PL) regression model fitting equation as: Y = −0.884 + (211.6 + 0.884)/[1 + (X/1.799)^(−0.7292)] (R^2^ = 0.9910, Figure 7A); while the detection sensitivity of NiV pseudovirus in pig serum reached 185 TU/mL, with its 4PL model equation as: Y = 1.273 + (79.84 − 1.273)/[1 + (X/4.608)^(−1.275)] (R^2^ = 0.9916, Figure 7B). In the human serum system, the LOD of NiV-G protein was further reduced to 24 pg/mL, with with its 4PL model equation as: Y = −0.2.310 + (432.3 + 2.310)/(1 + (X/1.847)^(−0.7317) (R^2^ = 0.9928, Figure 7C); the detection sensitivity of NiV pseudovirus also reached 185 TU/mL, with its 4PL model equation as: Y = 1.022 + (249 − 1.022)/(1 + (X/5.503)^(−1.035) (R^2^ = 0.9948, Figure 7D). The above data indicate that this detection method possesses high sensitivity and strong robustness (R^2^ > 0.99) across cross-species serum matrices, making it suitable for precise quantitative analysis of NiV pathogens in complex biological samples.

## 4. Discussion

Since the identification of Nipah virus (NiV) in 1999, a total of 647 confirmed cases have been reported in Southeast Asia, characterized by periodic outbreaks, with case fatality rates ranging from 40 to 75 percent [25,26]. Epidemiological studies have shown that human infection is mainly through the following routes: (1) direct ingestion of fruit or palm juice contaminated with saliva; (2) inhalation of virus-containing urine/saliva aerosols; and (3) exposure to contaminated animal products [27]. Close contact with a person with NiV in different settings (e.g., in a hospital) can lead to human-to-human transmission of NiV [28]. Given that there are no clinical therapeutics or prophylactic vaccines approved for NiV [29], early diagnosis remains the core strategy of epidemic control, and it can effectively interrupt the chain of transmission through timely identification of infected individuals, isolation, and symptomatic treatment.

The clinical differentiation of Nipah virus (NiV) infection poses significant diagnostic challenges due to its nonspecific symptomatology overlapping with other febrile illnesses [30]. Current laboratory approaches primarily employ molecular and serological methodologies, including reverse transcription-polymerase chain reaction (RT-PCR), genome sequencing, enzyme-linked immunosorbent assay (ELISA), plaque reduction neutralization testing (PRNT), indirect fluorescent antibody assay (IFA), histopathological examination, and viral culture techniques [31]. Among these, RT-PCR remains the diagnostic gold standard for acute-phase detection owing to its rapid turnaround time. However, this method presents operational limitations, including a 2–4 h procedural duration that increases human error potential, coupled with cold chain-dependent qPCR reagent requirements.

In contrast, the AlphaLISA detection platform demonstrates significant operational advantages with a 30 min assay time and enhanced reagent stability at 2–8 °C for six months, presenting critical utility for resource-limited settings. While initial equipment investment ranges from 2000 to 5000 (compared to RT-qPCRs 1000–2000), both systems maintain equivalent per-test costs ($5/sample). The AlphaLISA system’s modular design achieves 15% space efficiency improvement and 60% training time reduction through pipette-free operation, contrasting with RT-PCRs stringent power requirements. This system’s cold chain independence enhances emergency deployment feasibility in infrastructure-deficient regions.

Conventional confirmatory methods, including virus isolation, PRNT, and electron microscopy, necessitate biosafety level 4 (BSL-4) containment [32], posing barriers due to sophisticated facility requirements and prolonged processing times. Although loop-mediated isothermal amplification (LAMP) offers shorter turnaround than conventional PCR, it still requires nucleic acid purification and amplification steps. In contrast, AlphaLISA employs direct protein detection methodology that circumvents nucleic acid handling, simplifying workflow while reducing contamination risks through its homogeneous reaction format. This streamlined protocol combines simplified operational procedures with time-efficient performance and enhanced biosafety profiles, establishing AlphaLISA as an optimized solution for NiV surveillance in resource-constrained environments. The system’s technical features provide critical infrastructure support for rapid outbreak containment and equitable global health responses [33].

The single B cell antibody screening technology has made significant progress in recent years, demonstrating unique advantages compared to traditional hybridoma technology. This technology directly clones natural B cell antibody genes, avoiding the issue of low cell fusion efficiency, and theoretically allows for comprehensive sampling of the complete B cell receptor repertoire [12]. Its cross-species applicability significantly broadens the sources of antibodies, facilitating the construction of a more diverse antibody resource library. In addition, although this study systematically evaluates the diagnostic application value of recombinant expressed Nipah virus monoclonal antibodies, functional validations regarding their affinity characteristics and neutralization activities have not yet been conducted, which limits a comprehensive elucidation of the mechanism of action of the candidate antibody molecules. Future research will employ in vitro and in vivo experimental platforms, such as pseudovirus neutralization assays and surface plasmon resonance analysis, to systematically analyze the functional characteristics of the antibodies and their interaction mechanisms with viral targets.

This study established a platform for the selection of antigen-specific B cells based on multi-parameter flow sorting technology. By employing multi-color fluorescent labeling of mouse spleen cells, high-purity sorting of IgG + memory B cells was achieved. The experimental design utilized dual fluorescently labeled antigen probes to identify B cell populations specifically binding to the antigen through dual positive signals, effectively eliminating non-specific binding interference [34]. After obtaining the antibody variable region genes from sorted cells via single-cell RT-PCR, high-affinity NiV monoclonal antibodies with titers reaching 1:10^7^ can be produced within a week using the Expi293 transient expression system. This technological framework provides an efficient antibody development strategy to address outbreaks of emerging infectious diseases.

Based on the aforementioned technological platform, this study innovatively established the Nipah virus AlphaLISA detection system. This technology employs a homogeneous chemiluminescent sandwich immunoassay, which enhances detection sensitivity by 2–3 orders of magnitude compared to traditional ELISA, primarily due to the 60,000 singlet oxygen molecules generated by the donor microspheres triggering a cascade signal amplification effect [35]. The high-density antibody coating strategy on the microsphere surface can further optimize detection sensitivity [36]. This method enables objective interpretation through quantitative fluorescence signals, featuring advantages such as simplicity in operation and rapid detection (less than 30 min), making it particularly suitable for large-scale rapid testing during the onset of an epidemic. Existing research has confirmed that the AlphaLISA technology demonstrates excellent specificity and sensitivity in the detection of SARS-CoV-*2* and the Hepatitis C virus [37], providing technical validation for the construction of this system.

Given the prevalence of NiV outbreaks in resource-limited, densely populated regions [38], the detection system developed herein offers several practical advantages: (1) high sensitivity (detection limit reaching pg level) and specificity (S/N < 1 for ZIKV, IAV, IBV, JEV, CV); (2) strong resistance to matrix interference (can tolerate serum samples); and (3) ease of operation (can be implemented by trained grassroots personnel). In regions lacking the conditions for real-time quantitative PCR detection, this technology can achieve large-scale screening of patients at different stages of infection, providing technical support for population-based epidemiological prevention and control as well as individualized early intervention.

This study did not validate the performance of AlphaLISA in detecting Nipah virus using clinical samples, primarily due to the lack of epidemiological surveillance data and avenues for obtaining clinical samples of this virus within China. Although the NIV-G protein and pseudovirus system can preliminarily assess the sensitivity and specificity of the method and simulate certain matrix interferences found in clinical samples (such as proteins and lipid components), they cannot fully replicate the genomic complexity of the natural virus (such as strain variation and epigenetic modifications), which may lead to deviations in the performance parameters when applied to real samples. Furthermore, it should be noted that the current study only assessed methodological performance through internal validation, which may introduce experimental design biases (such as sampling selection and variations in operational standardization), lacking reproducibility verification in a multi-center environment. In the future, we plan to obtain clinically validated samples (such as blood and cerebrospinal fluid) through multi-center collaborations, covering cases of acute/recovery infections, different virus subtypes (such as genotypes I and III), and co-infection cases. This will systematically evaluate the method’s anti-interference capability and cross-platform comparability in real samples. Additionally, we are advancing the development of automated processes to reduce human operational errors and plan to collaborate with third-party laboratories to conduct independent validation, thereby enhancing the credibility and global applicability of the results.

## 5. Conclusions

This study successfully established a light-activated chemiluminescence detection system based on a single B cell antibody development platform and NiV glycoprotein (G protein) targeting recognition. Experimental data confirm that the AlphaLISA system exhibits significant advantages in sensitivity and time efficiency: its limit of detection (LoD) reaches 24 pg/mL (picogram level), enabling precise identification of low-abundance viral proteins in early infection samples. Furthermore, relying on a fully automated liquid handling platform (compatible with 96-well plates), the detection process can be completed in less than 30 min, showcasing both speed and throughput advantages. Compared to traditional ELISA (LoD typically ≥ 1 ng/mL) or real-time fluorescent PCR (which requires 2–4 h), this technology significantly reduces operational complexity and minimizes human error through a homogeneous reaction system and automated design, thus meeting the high-throughput screening demand in emergency detection during outbreaks (e.g., processing thousands of samples daily), providing a rapid and reliable laboratory-level solution for epidemic prevention and control. It demonstrates remarkable potential for epidemiological applications and may be expanded in the future as a monitoring tool for zoonotic diseases in Southeast Asia’s natural epidemic sources, especially in high-risk scenarios such as palm oil plantations and wildlife trade markets, where large-scale serological screening can assess the latent transmission risks of NiV. This proactive monitoring model will effectively address the lagging deficiencies of the current passive monitoring system, providing a technological paradigm for the early warning of novel zoonotic diseases.

## Figures and Tables

**Table 1:** Intra-assay and Inter-assay Precision.

Concentration (ng/mL)	Intra-Assay Mean		Inter-Assay Mean	
	$\bar{x}\pm s$	CV (%)	$\bar{x}\pm s$	CV (%)
10 ng/mL	84.75 ± 9.46	6.42	89.12 ± 5.89	6.14
1 ng/mL	13.66 ± 2.26	8.03	14.98 ± 1.26	7.94
0.1 ng/mL	2.55 ± 0.39	7.63	2.82 ± 0.26	9.50

## Data Availability

The data that support the findings of this study are available from the corresponding author upon reasonable request.

## Supplementary Materials

The following supporting information can be downloaded at: https://www.mdpi.com/article/10.3390/v17060748/s1, Table S1: Sequence of primer and size of product for antibody variable region amplification. Figure S1: Indirect ELISA dynamic analysis of antibody-antigen binding affinity.
