# Supplementary material for: A Highly Specific Antibody-Based Assay for Nipah Virus AlphaLISA Detection
Source: Viruses. 2025 May 23;17(6):748. doi: 10.3390/v17060748 (PMC12197547; doi:10.3390/v17060748)
Supplement: Supplementary file 1 [file viruses-17-00748-s001.zip › viruses-3581988-supplementary.pdf]

Table S1. Sequence of primer and size of product for antibody variable region amplification

Primer names	Primer sequences	
1mF-1	AGGAACTGCAGGTGTCC	Primary amplification primers
1mF-2	CAGCTACAGGTGTCCACTCC	
1mF-3	TGGCAGCARCAGCTACAGG	
1mF-4	CTGCCTGGTGACATTCCCA	
1mF-5	CCAAGCTGTGTCCTGTC	
1mF-6	TTTTAAAAGGTGTCCAGKGT	
1mF-7	CCTGTCAGTAACTRCAGGTGTCC	
1mF-8	TTTTAAAAGGGGTCCAGTGT	
1mF-9	ATGAAGTTGTGGYTRAACTGG	
1mF-10	TGTTGGGGCTKAAGTGGG	
1mF-11	CGTTCCTGGTATCCTGTCT	
1mRG	AGAAGGTGTGCACACCGCTGGAC	
1mFK-1	RGTGCAGATTTTCAGCTTCCTGCT	
1mFK-2	TGGACATGAGGGCYCCTGCTCAGT	
1mFK-3	CTSTGGTTGTCTGGTGTTGAYGGA	
1mFK-4	GTTGCTGCTGCTGTGGCTTACA	
1mFK-5	GTATCTGGTACCTGTGG	
1mFK-6	TGCCTGTTAGGCTGTTGGTGCT	
1mFK-7	GCTCAGTTCCTTGGTCTCCTGTTGC	
1mFK-8	TGGGTGCTGCTGCTCTGGGT	
1mFK-9	CAGTTCCTGTTTCTGTTARTGCTCTGG	
1mFK-10	TGCTCTGGTTATATGGTGCTGATGGG	
1mRK	ACTGAGGCACCTCCAGATGTT	
2FG	GGGAATTCGAGGTGCAGCTGCAGGAGTCTGG	Nested amplification primers
2RG	GCTCAGGGAARTAGCCCTTGAC	
2FK	GAYATTGTGMTSACMCARWCTMCA	
2RK	TGGGAAGATGGATACAGTT	

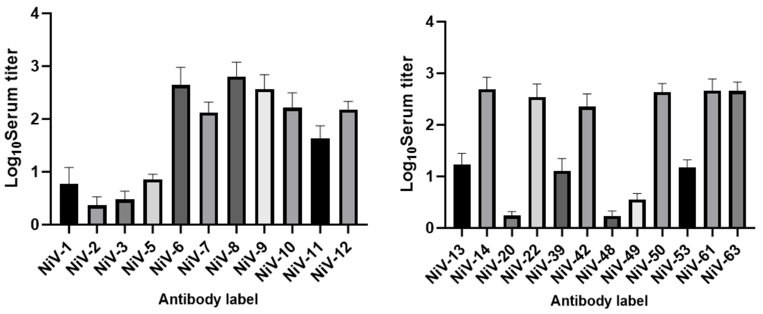

Figure S1 Indirect ELISA dynamic analysis of antibody-antigen binding affinity
